# Retrospective analysis of the incidence of appendiceal neoplasm and malignancy in patients treated for suspected acute appendicitis

**DOI:** 10.1186/s12893-024-02412-4

**Published:** 2024-04-24

**Authors:** Eliane Dohner, Fiona Joséphine Kierdorf, Rupert Langer, Markus Zuber, René Fahrner

**Affiliations:** 1 Department of Surgery, Bürgerspital Solothurn, Solothurn, Switzerland; 2 Department of Visceral Surgery and Medicine, Inselspital, Bern University Hospital, Bern, Switzerland; 3 Institute of Pathology and Molecular Pathology, Kepler University Hospital, Johannes Kepler University, Linz, Austria; 4 Clarunis University Center for Gastrointestinal and Liver Diseases, St. Clara Hospital Basel and University Hospital, Basel, Switzerland; 5 Department of Vascular Surgery, University Hospital Bern, University of Bern, Bern, Switzerland

**Keywords:** Acute appendicitis, Appendiceal neoplasm, Appendiceal malignancy, Appendectomy, Retrospective study

## Abstract

**Background:**

Nonoperative management of uncomplicated appendicitis is currently being promoted as treatment option, albeit 0.7–2.5% of appendectomies performed due to suspected acute appendicitis show histologically malignant findings. The purpose of this study was to investigate the incidence of neoplasm and malignancy of the appendix in patients presenting with suspected acute appendicitis in real world setting.

**Methods:**

This is a retrospective single-centre investigation of 457 patients undergoing appendectomy between the years 2017–2020. The patients’ demographics, symptoms and diagnosis, intraoperative findings, and histopathological results were analysed.

**Results:**

In 3.7% (*n* = 17) histological analysis revealed neoplasms or malignancies. Median age was 48 years (20–90 years), without sex predominance. Leukocytes (11.3 ± 3.7 G/l) and C-reactive protein (54.2 ± 69.0 mg/l) were elevated. Histological analysis revealed low-grade mucinous appendiceal neoplasia (*n* = 3), sessile serrated adenoma of the appendix (*n* = 3), neuroendocrine tumours (*n* = 7), appendiceal adenocarcinoma of intestinal type (*n* = 3), and goblet cell carcinoma (*n* = 1). Additional treatment varied between no treatment or follow-up due to early tumour stage (*n* = 4), follow-up care (*n* = 3), additional surgical treatment (*n* = 8), or best supportive care (*n* = 2).

**Conclusions:**

Preoperative diagnosis of appendiceal tumours is difficult. Nonoperative management of patients with acute, uncomplicated appendicitis potentially prevents the correct diagnosis of malignant appendiceal pathologies. Therefore, close follow-up or surgical removal of the appendix is mandatory.

## Background

Appendicitis is one of the most common acute digestive diseases, with an incidence of 110–200/100,000 persons per year [1, 2]. The standard therapy is appendectomy, which is mostly performed using laparoscopic techniques [1, 3–7]. However, in cases of acute, uncomplicated appendicitis, nonoperative management is currently being suggested as a possible treatment alternative to surgery in selected patients [8–14]. However, 0.7–2.5% of cases with suspected acute appendicitis show histologically premalignant or malignant findings [15–19]. A non-surgical management option may thus lead to a delay in diagnosis and appropriate treatment [20]. Furthermore, there are some studies showing an increased rate of malignant findings in interval appendectomies than in acute surgery [21, 22].

Most of the patients with tumours present with clinical symptoms comparable to acute appendicitis, which may be caused by tumour induced local inflammation or a chronic inflammatory state of the appendix leading to tumour formation. Symptoms such as weight loss, pain and abdominal distension are present in advanced disease due to peritoneal dissemination [23]. The classification of primary appendiceal cancer is usually performed according to the WHO classification, but alternative nomenclatures are also used worldwide [23]. Specific staging systems and treatment guidelines exist for some subtypes (e.g., mucinous or neuroendocrine neoplasms), but for other entities (e.g., adenocarcinoma), there are only guidelines that are extrapolated from colorectal cancer because of the lack of randomised, prospective trials [23]. Histological diagnosis is usually performed by morphology using haematoxylin-eosin (H&E) routine staining but can be supplemented by immunohistochemical staining [24].

There are several risk factors reported for underlying malignant disease of the appendix that should be considered in patients with suspected acute appendicitis: age, multiple comorbidities, atypical presentation, complicated appendicitis, absence of leucocytosis, and diameter of the appendix on ultrasound of > 13 mm [15, 19, 25].

In Switzerland ultrasound and abdominal CT are regularly used diagnostic tools beside of the patient’s history and clinical examination. Especially in older patients, females and not obvious signs for acute appendicitis, radiological imaging is commonly used. Although, the radiological findings are often unspecific, it may help to avoid negative appendectomy. The differentiation of acute appendicitis and small appendiceal tumours is often difficult to distinguish.

Radiological features for appendiceal tumours in ultrasound and computed tomography (CT) are nonspecific and occult [26, 27]. But depending on the entity of appendiceal malignancies some sonographic features such as enlarged or small inner luminal diameter, thick and irregular appendix wall, loss of layer pattern of the appendix wall, and even hypoechogenicity are described [26]. In addition, infiltration of the periappendiceal fat or even abscess formation with suspicion of appendix perforation may be seen in cases of appendiceal malignancies [26]. Findings in abdominal CT scans are again depending on the underlying type of malignancy and reach from hyperenhancement, nodular wall thickening, calcification, mucocele, lymphadenopathy, periappendiceal fat infiltration, or tumour mass [27].

The purpose of this retrospective study was to investigate the incidence and clinical characteristics of patients who presented with suspected acute appendicitis and were finally diagnosed neoplasm or malignancy of the appendix.

## Methods

This retrospective single-centre investigation included all patients with suspected acute appendicitis who underwent an appendectomy between 10/2017 and 05/2020 in a Swiss cantonal hospital. Inclusion criteria were clinical suspicion of acute appendicitis, age over 18 years, and surgical treatment. Patients undergoing incidental appendectomy during other surgical procedures were excluded from this analysis. Overall, 457 patients were included in this investigation.

All operations were intended as laparoscopic procedure using a three port technique. A conversion to open surgery was necessary because of extended surgery, adhesions or inflammatory changes with unclear anatomic situation. Laparoscopic appendectomy is mostly performed by resident surgeons in attendance with an experienced staff surgeon.

From the clinical patients’ records, the following parameters were collected: patient characteristics (age, sex, body mass index (BMI), American Society of Anaesthesiologists (ASA) score, presence of another diagnosis), symptoms and clinical signs of acute appendicitis (right lower quadrant pain, duration of pain, inappetence, shivering, temperature, positive McBurney sign, guarding, rebound tenderness, positive psoas sign), diagnostic tests (blood test, ultrasound, CT scan, histology), duration of operation and postoperative complications, as well as the use of antibiotics postoperatively. In addition, the treatment and course of the patients with appendiceal tumours was recorded. The histological analysis was performed morphologically using routine H&E staining and in some selected patients with immunohistochemical staining. Based on the intraoperative and histological diagnosis, two groups (with and without malignancy in patients presenting with symptoms of acute appendicitis) were compared regarding the assessed parameters.

## Ethics

This retrospective study was approved by the Ethics Committee of Northwest and Central Switzerland (EKNZ No. 2020 − 01592) and due to the retrospective nature of the study, the need for informed consent was waived by the Ethics Committee. In addition, the investigation was carried out in accordance with the current version of the Declaration of Helsinki and conformed to national legal and regulatory requirements.

## Statistics

Continuous variables are reported as the mean and standard deviation or median and interquartile range as appropriate. The groups were compared using Student’s t test or Mann‒Whitney U test as indicated. Categorical variables are reported as numbers and proportions and were compared with the χ2 and Fisher’s exact test. GraphPad Prism 5.0 software package (GraphPad, San Diego, California, USA) was used for statistical analysis of the data and graphics, and a p value ≤ 0.05 was defined as statistically significant.

## Results

In the majority of patients, the assumed preoperative diagnosis of acute appendicitis was confirmed during surgery (*n* = 385, 84.2%). In all patients, the appendix was removed as intended. In 12.1% (*n* = 55) of patients non-inflamed appendix or other non-tumorous pathologies were identified (endometriosis, fibrous obliteration, neurogenic appendicopathy, oxyuriasis, presence of Meckel’s diverticulum, urachus fistula). In 17 patients (3.7%), histological analysis revealed neoplasm or malignancy. There were no statistically significant differences for age, sex, BMI, ASA score, or preexisting diseases (Table 1). The clinical presentation of patients with and without tumours did not differ significantly regarding the localisation and duration of pain, inappetence, shivering, guarding, or rebound tenderness (Table 2). A positive McBurney sign was more frequently reported in patients without tumours (without tumours *n* = 403, 91.6% vs. with tumours *n* = 13, 76.5%; *p* = 0.032, Table 2).

**Table 1:** Patients’ characteristics

	With tumour	Without tumour	*p* value
Age (years) ^a^	47.8 ± 18.8	40.8 ± 17.1	0.1 ^d^
Sex, ratio female : male ^b^	52.9% : 47.1%	42.7% : 47.3%	0.986 ^e^
BMI (kg/m^2^) ^a^	27.2 ± 7.0	26.2 ± 6.3	0.45 ^d^
Preexisting other diagnosis ^b^	10 (58.8%)	298 (67.7%)	0.442 ^e^
ASA-Score ^c^	2 (1–4)	2 (1–4)	0.257 ^e^

BMI: body mass index; ASA-Score: American Society of Anaesthesiologists

^a^Mean values ± standard deviation

^b^Values are numbers (% of all patients in the group)

^c^Median values with range

^d^Student’s t test

^e^Pearson’s chi square test

**Table 2:** Clinical presentation of patients with and without appendiceal tumours

	With tumour	Without tumour	*p* value
Duration of pain (hours) ^a^	71.6 ± 116.4	54.1 ± 92.1	0.445 ^c^
Right lower quadrant pain ^b^	15 (88.2%)	415 (94.3%)	0.297 ^d^
Inappetence ^b^	9 (52.9%)	208 (47.3%)	0.646 ^d^
Shivering ^b^	0	63 (14.3%)	0.093 ^d^
Body temperature (°C) ^a^	36.7 ± 0.8	36.9 ± 0.8	0.304 ^c^
McBurney sign ^b^	13 (76.5%)	403 (91.6%)	0.032 ^d^
Guarding ^b^	4 (23.5%)	166 (37.7%)	0.235 ^d^
Rebound tenderness ^b^	7 (41.2%)	253 (57.5%)	0.182 ^d^
Psoas sign ^b^	2 (11.8%)	88 (20%)	0.665 ^d^

^a^Mean values ± standard deviation

^b^Values are numbers (% of all patients in the group)

^c^Student’s t test

^d^Pearson’s chi square test

Leucocytes and C-reactive protein (CRP) levels were elevated in both groups but showed no statistically significant differences (leucocytes: without tumours 12.8 ± 4.6 vs. with tumours 11.3 ± 3.7 G/l; *p* = 0.177; CRP: without tumours 57.7 ± 70.0 vs. with tumours 54.2 ± 69.0 mg/l; *p* = 0.838). Ultrasound and CT scan were not routinely used in either group and were only performed in selected patients. Interestingly, sonography was less often and CT scans were more often used in patients with tumours than in patients without tumours, but again, there were no statistically significant differences (ultrasound: without tumours *n* = 245 (55.7) vs. with tumours *n* = 8 (47.1%); *p* = 0.483; CT scan: without tumours *n* = 179 (40.7%) vs. with tumours *n* = 9 (52.9%); *p* = 0.313).

Based on symptoms and clinical or diagnostic findings, appendiceal tumour was only suspected preoperatively in two patients. Almost all patients showed intraoperative signs of appendiceal inflammation (16/17 patients). There was no difference in the rate of complicated appendicitis between the two groups. Postoperative antibiotics were statistically significantly more frequently administered in patients with tumours (*n* = 10, 58.8% vs. *n* = 131, 29.8%; *p* = 0.01), without influencing the rate of postoperative complications (Table 3). The rate of conversion to open surgery was significantly higher in patients with tumours (*n* = 3, 17.6% vs. *n* = 2, 0.45%; *p* < 0.0001), and there was a higher incidence of extended surgery, such as right hemicolectomy during the initial operation (*n* = 2, 11.8% vs. *n* = 0; p = < 0.0001), leading to a statistically significant longer duration of operation (114 min vs. 80 min; *p* < 0.0001).

**Table 3:** Operative and postoperative data in patients with and without appendiceal tumours

	With tumour	Without tumour	*p* value
Operation time (min) ^a^	113.6 ± 55.5	79.7 ± 29.4	< 0.0001 ^c^
Complicated appendicitis ^b^	10 (58.8%)	194 (44.1%)	0.231 ^d^
Conversion to open surgery ^b^	3 (17.6%)	2 (0.45%)	< 0.0001 ^d^
Postoperative complications ^b^	2 (11.8%)	28 (6.3%)	0.378 ^d^
Antibiotics postoperatively ^b^	10 (58.8%)	131 (29.8%)	0.011 ^d^

^a^Mean values ± Standard deviation

^b^Values are numbers (% of all patients of the group)

^c^Student’s t-test

^d^Pearson’s chi square test

Histological analysis of the appendix revealed the following tumour entities: low-grade mucinous appendiceal neoplasia (LAMN, *n* = 3, 17.6% of all tumourous cases), sessile serrated adenoma of the appendix (*n* = 3, 17.6% of all tumourous cases), neuroendocrine tumours (*n* = 7, 41.2% of all tumourous cases), appendiceal adenocarcinoma of intestinal type (*n* = 3, 17.6% of all tumourous cases), and goblet cell carcinoma (*n* = 1, 6% of all tumourous cases). After subsequent tumour staging, eight patients underwent additional surgical treatment, including diagnostic laparoscopy with biopsies (*n* = 4) and right hemicolectomy (*n* = 3), and in one patient, partial omental resection and peritonectomy were necessary after simultaneously performing right hemicolectomy due to a neuroendocrine tumour. Surveillance was selected for three patients, whereas for four patients, no further surveillance or treatment was considered necessary due to small tumours or tumour entities. Two patients had poor physical condition, so no additional treatment was initiated (Table 4).

**Table 4:** Pathologic examination, stage and follow-up and adjuvant therapy

Pathology	Age (years)	Mean age	Sex	Incidence	Stage	Diagnostics during follow-up	Adjuvant therapy
Low-grade mucinous appendiceal neoplasia (LAMN, *n* = 3)	32	51.3	M	0.7%	pTis R0	sonography after 6 months	
	62		F		pT4a pM0, Stadium IIB	diagnostic laparoscopy	
	60		F		pTis	colonoscopy and diagnostic laparoscopy	
Sessile serrated adenoma of the appendix (*n* = 3)	28	35.7	F	0.7%	no dysplasia	no follow-up	
	39		F		no dysplasia	no follow-up	
	40		F		no dysplasia	colonoscopy	
Neuroendocrine tumours (*n* = 7)	20	39.3	M	1.5%	pT3 pNx V0 L0 Pn0 G1 R1		right hemicolectomy
	41		M		pT3a L0 V0, G1		right hemicolectomy
	27		M		pT4 L1 V0 Pn0 RX	DOTATATE-PET-CT, gastroscopy, colonoscopy, diagnostic laparoscopy and biopsy	
	50		M		pT4 pN2 (4/17) pM1b (PER) L1 V0 Pn0 G1 R0	DOTATATE-PET-CT	simultaneous right hemicolectomy, partial resection omentum majus, peritonectomy, palliative treatment
	24		M		pT1 pNx L0 V0 Pn0 G1 R0	DOTATATE-PET-CT	
	52		F		pT1 pNx L0 V0 Pn0 G1 R0	no follow-up	
	61		M		pT1 pNx L0 V0	no follow-up	
Appendiceal adenocarcinoma from intestinal type (*n* = 3)	90	74.0	F	0.7%	pT4b pN1b (2/17) L1 V0 Pn0 G2 R1	Exitus letalis (septic shock)	simultaneous right hemicolectomy
	69		F		pT3 pNx L0 V0 Pn0 G2 R1		palliative chemotherapy
	63		M		pT4a pNx L1 V0 Pn1 G2 R0		hemicolectomy, lymphadenectomy and adjuvant chemotherapy proposed, but not applied due to poor general condition and patient refusal
Goblet cell carcinoma (*n* = 1)	54		F		pT3 pN0 (0/10) L0 V0 Pn1 G2 R0	CT-scan and colonoscopy	right hemicolectomy a few weeks later, bilateral ovarectomy during follow-up

## Discussion

In this retrospective single-centre investigation, the rate of appendiceal malignancy or neoplasm was 3.7%, and thus higher than in previously reported studies (0.7–2.5%) [15–19], which caused further analysis. A specific feature to explain the higher tumour incidence in our institution was, that in all patients suspected for acute appendicitis, surgical treatment was the primary treatment option, and even macroscopically unsuspicious appendices were removed. This approach to remove macroscopically normal-appearing appendices is controversial. Van den Broek et al. stated that it would be safe not to perform an appendectomy even if no other source of pain is found during surgery [28]. In contrast, Roberts et al. demonstrated that surgeons’ ability to diagnose and differentiate a normal appendix is low, and even malignant lesions may be missed [29]. Whether this approach is also responsible for the comparatively higher rate of malignant lesions is debatable, furthermore the increased use of imaging modalities has been postulated to be responsible for the increase rate of appendiceal malignancies [30], whereas in this investigation a definite preoperative diagnosis was only possible in a few of patients.

Nonoperative management is currently being promoted as a feasible treatment option for patients with acute, uncomplicated appendicitis and 65% of patients are symptom-free after one year [31]. However, there are some reports showing that the rate of appendiceal and colonic malignancies is increased in interval appendectomies in comparison with the rate of malignancies in appendectomies performed in the acute state [21, 32]. Possible explanations for this effect may be that the initial symptoms of appendicitis are caused by a local inflammatory tumour effect or that malignant transformation is caused by a chronic inflammatory state [29, 33]. Both effects may further explain the high blood inflammation markers and macroscopically inflamed appendices during surgery.

In previous studies, several risk factors for potential appendiceal malignancy were reported: age ≥ 50 years, atypical symptoms, presence of appendiceal phlegmon [19] or dilatation of the appendix [25] in radiological findings, immunosuppressive therapy including the use of steroids [19, 34], absence of leucocytosis [25], and elevated CRP ≥ 54 mg/l [15]. Furthermore, female sex [28], ASA score ≥ 2, Crohn’s disease, anaemia on admission [15, 19] and a history of previous malignancy [19] were found to be risk factors. Unfortunately, these risk factors are not particularly sensitive or specific in the cohort of patients with suspected acute appendicitis. In the present study, none of the previously reported risk factors showed statistically significant differences between patients with and without tumours. In addition to the low specificity and sensitivity of the mentioned risk factors, the small number of patients and the heterogeneity of tumour entities lead to potentially different clinical symptoms.

Appendiceal neoplasms and malignant tumours are a heterogeneous group of diseases [23]. Less frequent tumours such as lymphoma, paraganglioma, metastasis, neuroma or leiomyoma may involve the appendix [2, 35], whereas neuroendocrine neoplasms (NEN) are the most frequent tumours of the appendix [28, 36], which is consistent with our data. NEN are often functional and produce hormones and systemic effects are seen depending on their size and possible hepatic metastasis, and include cutaneous flushing, hypotension, bronchoconstriction, diarrhoea, and right-sided cardiac valvular fibrosis [32, 37].

Most NEN are located at the tip of the appendix, but NEN located in the base have a higher risk for incomplete excision or recurrence [36]. Depending on the diameter and risk of lymph node infiltration oncological right hemicolectomy is recommended [35, 36, 38], or even cytoreductive surgery and hyperthermic intraperitoneal chemotherapy is necessary [39]. In this study, the majority of patients with NEN/NET in the appendix had an advanced tumour stage (T3 or T4), so extended surgical treatment, including right hemicolectomy, peritonectomy, or at least additional diagnostic laparoscopy, was necessary.

Nonmucinous adenocarcinoma is a very rare tumour entity and its evolution is comparable to the adenoma-carcinoma sequence in colorectal adenocarcinoma [23]. Currently there is no specific treatment recommendation, so the therapy is comparable to the workup, staging and treatment of colon cancer [23]. In the present study, two patients were found to have nonmucinous adenocarcinoma, and both had an advanced tumour stage, leading to extended surgery and palliative chemotherapy.

The group of mucinous neoplasms, also called cystadenomas, or mucinous adenocarcinomas (cystadenocarcinomas) are divided into noninvasive, low-grade appendiceal mucinous neoplasms (LAMN) or high-grade appendiceal mucinous neoplasms (HAMN) depending on the degree of cellular atypia [35], and are often associated with synchronous colorectal adenocarcinoma [23]. A rupture of epithelial cancerous cells may lead to intraperitoneal and distant extension of these tumorous cells (“pseudomyxoma peritonei”) [35]. LAMN are often cured with a simple appendectomy [35], for invasive mucinous adenocarcinomas an oncologic right hemicolectomy should be considered due to high risk of lymph node or intraperitoneal dissemination [23]. The treatment of pseudomyxoma peritonei includes complete cytoreductive surgery (CRS) and hyperthermic intraperitoneal chemotherapy (HIPEC), and when untreated, it invariably results in death [40].

Goblet cell carcinoma (GCC) is a rare entity, accounting for 14–19% of all primary appendix tumours, and is a mixed epithelial (glandular) and neuroendocrine neoplasm containing goblet cells [23]. Tang et al. proposed a classification (group A - C) based on histology, arrangement of the goblet cells, the degree of atypia, and desmoplasia with specific risks for metastases and poor outcome [24]. Based on these factors the management of patients differ between additional surgery (hemicolectomy, CRS and peritonectomy, or adjuvant treatment). In our cohort, GCC of the appendix was confirmed in only one female patient, and a right hemicolectomy and local peritonectomy were performed three weeks after the initial treatment. Comparable to previous reports with tumorous infiltration of the peritoneum, omentum and ovarian metastasis [24], a suspicious ovarian lesion was detected during follow-up in our female patient. The surgical exploration with ovarectomy revealed no tumour detection. Due to rare data treatment recommendations are limited, but localised (pT1 and pT2) typical GCC may be managed with simple appendectomy and close follow-up, while extended tumours should be treated with right hemicolectomy [23] and chemotherapy to avoid peritoneal spreading [23, 36].

The incidence of sessile serrated adenomas is estimated in one out of 1100 appendectomy specimens and the majority is found incidentally [41]. It is postulated that these lesions reveal in contrast to colorectal serrated lesions high rates of KRAS and lower rates of BRAF 500 mutations [34]. Serrated lesions of the appendix are limited to the muscularis mucosae and lamina propria, so that a complete resection for treatment is sufficient as performed in the underlying investigation [34]. A routine colonoscopy to rule out other lesions in the colon should be performed.

In this study, due to limited value of clinical risk factors, and radiological findings, malignancy was only suspected in two patients during the preoperative workup which is comparable to previous results [19]. Therefore, it is crucial that a histological workup is performed for all patients undergoing appendectomy [34]. Furthermore, close follow-up should be performed for patients undergoing nonoperative treatment in cases of suspected uncomplicated and complicated appendicitis to rule out a malignant process. A recent study revealed a positive association of complicated appendicitis and malignancy, and therefore suggested close follow-up and need for interval appendectomy [42]. Colonoscopy should be performed in cases of appendiceal tumour detection to exclude simultaneous colorectal cancer, which has been described in the literature in up to 18% of cases [36]. In this study, a postoperative colonoscopy was only performed in a few patients, without additional tumour detection.

Surgery due to appendiceal tumour was associated with increased surgical extension, and therefore led to a higher rate of surgical conversion, additional surgical procedures, and statistically significant longer operation time. Brunner et al. also showed a higher rate of open surgery in patients treated for appendiceal tumours [15].

Limitations of this investigation are the retrospective and nonrandomised nature of the data collection, no structured long-term follow-up regarding the disappearance of abdominal pain, and lack of data about patients with potential appendicitis treated without surgery. Furthermore, the small sample size of the investigated patient cohort and of each tumour entity makes it impossible to draw any valid conclusions or even treatment algorithms. To obtain valid data a multi-centric and international registry analysis would be necessary to further elucidate elementary treatment issues.

## Conclusions

In conclusion, nonoperative management of patients presenting with acute, uncomplicated appendicitis is currently described as an adequate treatment option. This potentially precludes a correct diagnosis of malignant appendiceal pathologies, especially since their clinical presentation, patient history, and intraoperative aspects may not differ significantly from acute appendicitis. Therefore, it is imperative to follow patients with nonoperative treatment or remove the appendix during the index admission to ensure further histological analysis. Nevertheless, primary appendix cancer is rare and is most often found incidentally during appendectomy or histology. There are many subtypes that can be broadly classified as colonic-type adenocarcinoma, mucinous neoplasm, goblet cell carcinoma and neuroendocrine neoplasm. Adequate treatment depends on the subtype, grading, and staging. Its treatment may range from appendectomy, oncologic hemicolectomy, and lymph node dissection to CRS and HIPEC with or without systemic chemotherapy.

## Data Availability

The datasets used and/or analyzed during the current study are available from the corresponding author on reasonable request.

## Abbreviations

ASA: American Society of Anaesthesiologists
BMI: body mass index
CRP: C-reactive protein
CRS: cytoreductive surgery
CT: computed tomography
HAMN: high-grade appendiceal mucinous neoplasms
HIPEC: hyperthermic intraperitoneal chemotherapy
GCC: goblet cell carcinoma
LAMN: low-grade appendiceal mucinous neoplasms
NEN: neuroendocrine neoplasms
